# Therapeutic education for empowerment and engagement in patients with Parkinson’s disease: A non-pharmacological, interventional, multicentric, randomized controlled trial

**DOI:** 10.3389/fneur.2023.1167685

**Published:** 2023-04-18

**Authors:** Maria Francesca De Pandis, Margherita Torti, Rossella Rotondo, Lanfranco Iodice, Maria Levi Della Vida, Miriam Casali, Laura Vacca, Fabio Viselli, Valeria Servodidio, Stefania Proietti, Fabrizio Stocchi

**Affiliations:** ^1^San Raffaele Cassino, Cassino, Italy; ^2^IRCCS San Raffaele Roma, Rome, Italy; ^3^Health Management, University Hospital “Federico II”, Naples, Italy; ^4^Italian Health Ministry c/o USMAF Campania, Naples, Italy; ^5^San Giovanni Battista Hospital, Rome, Italy; ^6^San Raffaele University, Rome, Italy

**Keywords:** patient education program, Parkinson’s disease, empowerment, motor fluctuations, rehabilitation, caregivers

## Abstract

**Background:**

In 1997 the European Parkinson’s Disease Associations launched the Charter for People with Parkinson’s disease that stated the right of patients to be informed and trained on the disease, its course, and treatments available. To date, few data analyzed the effectiveness of education program on motor and non-motor symptoms of PD.

**Objective:**

The aim of this study was to evaluate the efficacy of an education program as it was a pharmacological treatment, thus choosing as the primary endpoint the change in daily OFF hours, the most widely used outcome in pharmaceutical clinical trials on PD patients with motor fluctuations. Secondary outcomes were change in motor and non-motor symptoms, quality of life and social functioning. The long-term efficacy of the education therapy was also evaluated by analyzing data collected at 12- and 24-weeks follow-up outpatient visits.

**Methods:**

One hundred and twenty advanced patients and their caregivers were assigned to the intervention or control group in a single-blind, multicentric, prospective, randomized study evaluating an education program structured in individual and group sessions over a 6-weeks period.

At the end of study, the intervention group showed a significant reduction in daily OFF hours compared to control patients (−1.07 ± 0.78 vs. 0.09 ± 0.35, *p* < 0.0001) and a significant improvement was also reported in most secondary outcomes. Patients retained significant medication adherence and daily OFF hours reduction at 12- and 24-weeks follow-up.

**Conclusion:**

The results obtained demonstrated that education programs may translate in a notable improvement in motor fluctuations and non-motor symptoms in advanced PD patients.

**Clinical Trial Registration:**Clinicaltrials.gov, identifier NCT04378127.

## 1. Introduction

Parkinson’s disease (PD) is a progressive neurodegenerative disorder characterized by motor and non-motor symptoms, which contribute to the burden of the disease to patients and their caregivers (1, 2). A multidisciplinary and comprehensive approach, based on the chronic care model, is considered the best way to manage motor and non-motor symptoms of the disease (3). Among the key aspects of the chronic care model are patient-centered care, patient engagement and empowerment, and health literacy.

Patient-centered care focuses on “providing care that is respectful of, and responsive to, individual patient preferences, needs and values, and ensuring that patient values guide all clinical decisions” (4). Patient engagement has been defined as the “process of building the capacity of patients, families, carers, as well as health care providers, to facilitate and support the active involvement of patients in their own care, in order to enhance safety, quality and people-centeredness of health care service delivery” (5). Patient empowerment “helps patients gain control over their lives, increasing their capacity to act on issues that they themselves define as important” (6). Health literacy has been defined “as the ability to obtain and understand health information in order to make informed decisions regarding health care” (7, 8).

In 1997, based on this model, the European Parkinson’s Disease Associations (EPDA) published the Charter for People with Parkinson’s disease, that enshrines the right of patients to be informed and trained on the disease, its course and treatments available, for the purpose of favoring the active and conscious involvement of the patient and caregivers in decisions concerning the management of illness.

To evaluate whether the introduction of Charter for People with PD has influenced the disease management for PD across Europe since it was introduced, in 2010 the Move for Change (MfC) campaign was launched. It consisted of a series of three pan-European surveys to identify the strengths and weaknesses of PD treatment and to determine the patient’s perspectives on the quality of disease management (9–11). Despite this request and the clear need expressed by patients’ associations for education programs (9, 12), few studies have been performed so far to evaluate the impact of education therapy in PD. Even if there are data in favor of the effectiveness of this approach on motor and non-motor symptoms, almost all studies focused on quality of life (QoL) as primary endpoint (13–17). Based on these premises, we designed a study to evaluate the efficacy of an education program as pharmacological treatment, thus choosing as the primary endpoint the change from baseline in mean daily OFF hours, the most widely used outcome in pharmaceutical clinical trials on PD patients with motor fluctuations.

The long-term efficacy of education therapy on daily OFF hours, therapy adherence, motor and non-motor symptoms of PD patients and caregivers’ burden was evaluated by analyzing the data collected during outpatient visits at 12 and 24 weeks.

## 2. Materials and methods

### 2.1. Ethics and trial design

This was a single-blind, multicentric, prospective, randomized study in which a total of 120 patients, receiving standard neurological and physical care, were assigned to two groups: an intervention group, who underwent a structured education program, and a parallel controlled group.

The trial was conducted according to the Declaration of Helsinki (October 1996) and to the International Conference on Harmonization (ICH) Guidelines on GCP (CPMP 135/95). The study has been registered at: clinicaltrials.gov, NCT04378127^1^ with the alternative name of MisterParkinson. Signed informed consent was obtained from each patient and caregiver before any study procedure, after approval by each local institutional IRBs/IECs (San Raffaele Ethic Committee and ASL Rome II Ethic Committee).

### 2.2. Participants

Participants were recruited between May 2015 to December 2018 in three multidisciplinary PD Centers located at the IRCCS San Raffaele Roma, at the San Raffaele Cassino and at the San Giovanni Battista Hospital in Rome, Italy,

Inclusion criteria were idiopathic Parkinson’s disease [according to United Kingdom Brain Bank criteria (18)] complicated by motor fluctuations with at least 1.5 h of daily OFF time, being aged 20–80 years, being able to complete questionnaires, having no severe cognitive impairment (Mini-Mental State Examination ≥ 24), and having a stable caregiver.

Exclusion criteria were atypical parkinsonian syndromes, being wheelchair bound and severe comorbidity significantly interfering with quality of life.

### 2.3. Sample size calculation

Sample size was calculated on the basis that there was an expected improvement in the time spent in OFF in at least 50% of the patients treated (OR 1.5) compared to the control group. Considering an allocation ratio of 1, and setting the error to 0.05, a selection of 120 patients (60 in the treatment group and 60 in the control group) returned a statistical power of 84.6%.

### 2.4. Randomization and blinding

The random allocation of patients to the intervention or the control group was performed before baseline assessments and was managed centrally, according to an automatically generated randomization list by an allocation ratio of 1:1. For each randomization number, a sealed envelope containing the randomization code was prepared by the data manager, who generated the randomization list. A research assistant, without involvement in the trial, assigned patients to each group according to randomization code, organized visits, and managed data entry.

The research staff that performed baseline and follow-up assessments, composed of a neurologist and a neuropsychologist for each investigational center, was unaware of group assignment and was kept blinded for the whole length of the study. Patients in both groups were asked to not share their assignment with any of the raters.

### 2.5. Intervention

The whole education program, named “School of Parkinson,” consisted of 6 meetings, 7-days apart, for a total of 6 weeks.

**Figure 1:**
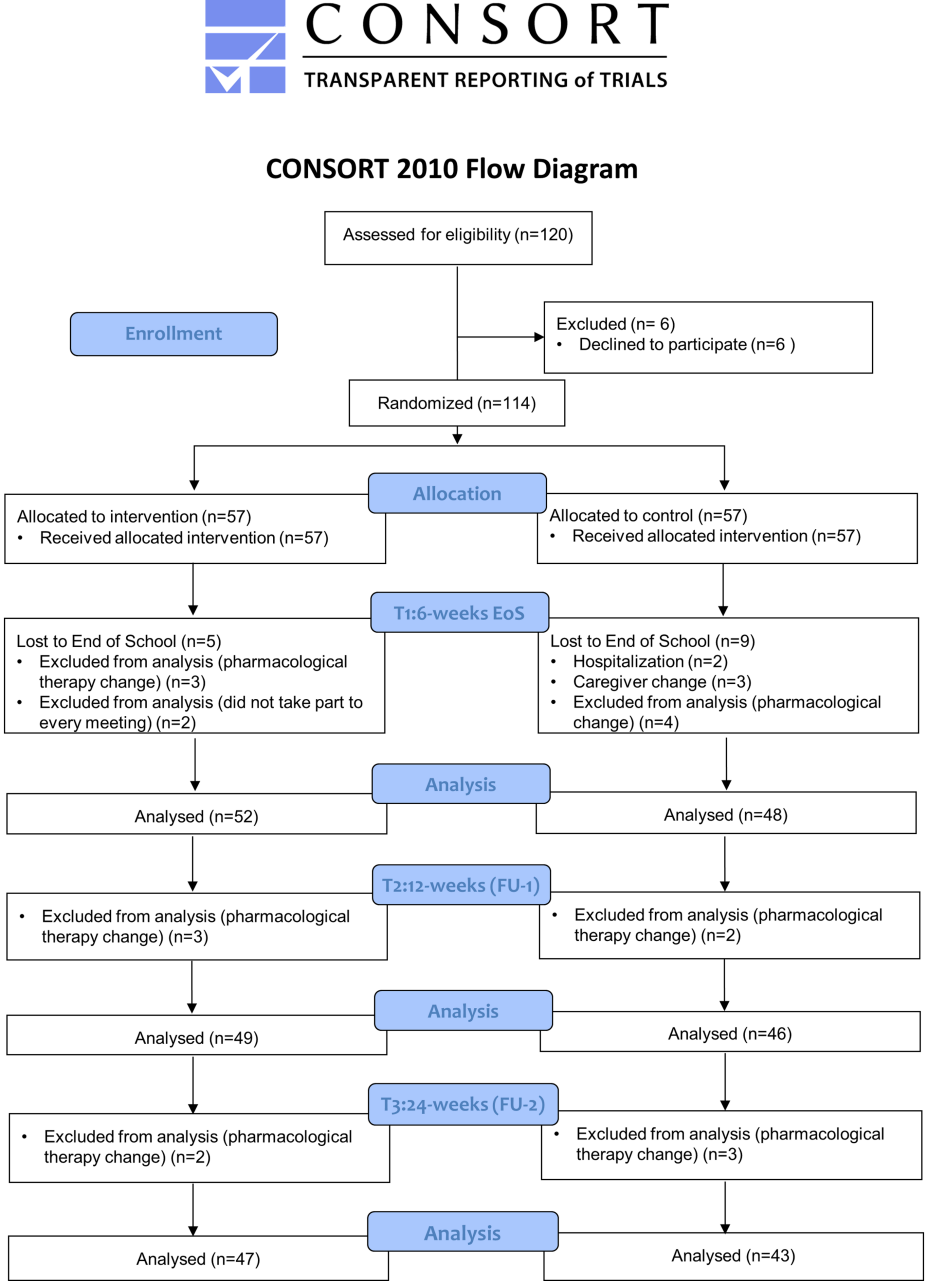
CONSORT 2010 flow diagram of the study.

Clinical evaluations of enrolled patients, lasting about 2 h, were scheduled within the 7 days prior the beginning of the program (T0: Baseline), at the conclusion of the education intervention (within the following 7 days; T1: 6 weeks—End of School), after 12 weeks (T2: 12 weeks FU1) and 24 weeks (T3: 24 weeks FU2) from the end of the program. The CONSORT diagram is shown in Figure 1.

**Figure 2:**
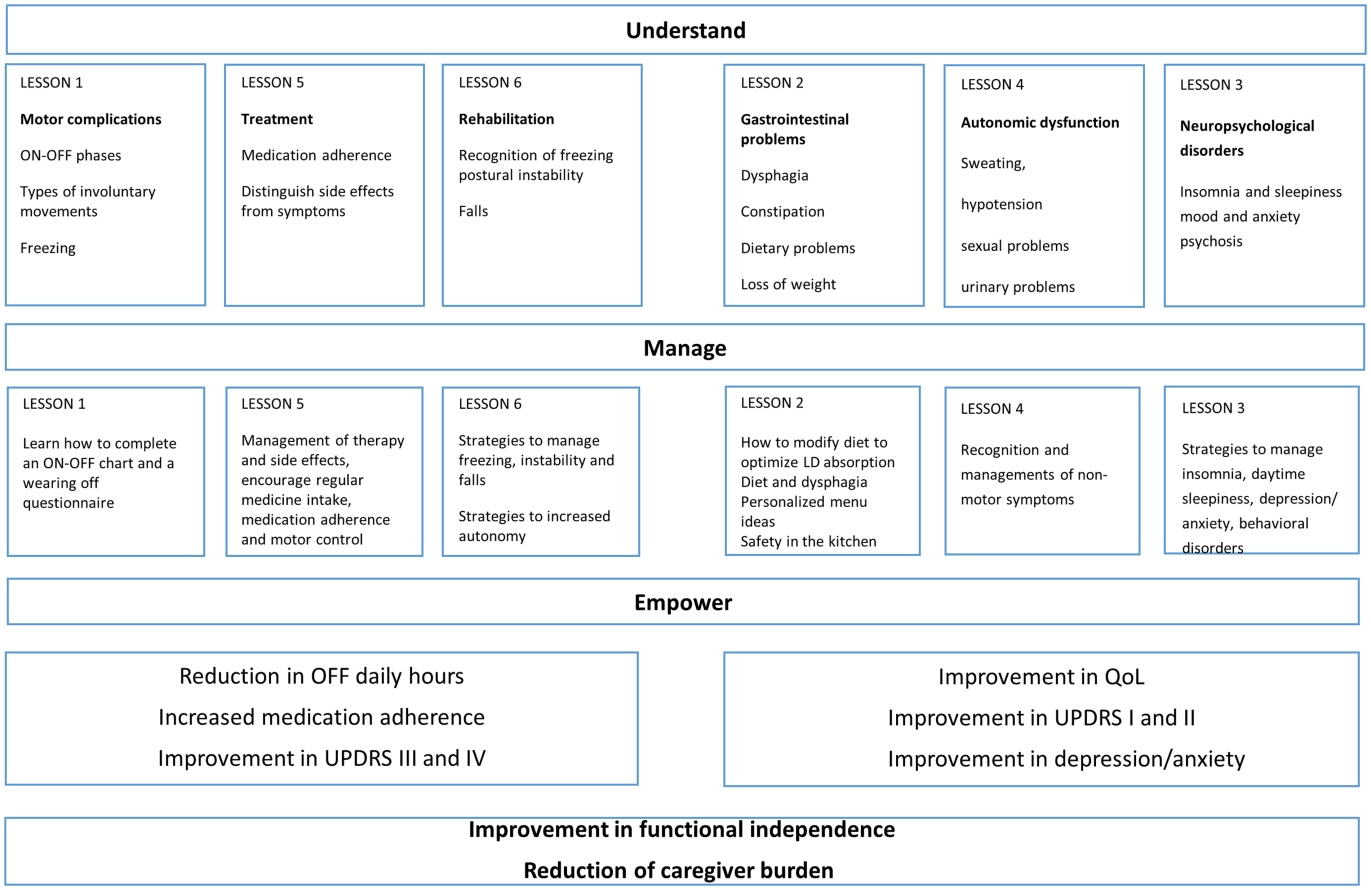
Diagram with topics of the six sessions of Parkinson’s disease (PD) educational program: in the diagram are illustrated the topics of the six sessions, divided in motor and non-motor outcomes. The aim of the program was to increase understanding of the disease, guide self-management, empower patients and caregivers and increasing their capacity to act on disease issues.

In addition to the baseline assessment, during the first meeting patient and caregiver received information about the disease; clarifications and advices were also provided on therapy related issues, as well as on motor and non-motor symptoms. Part of the meeting was also dedicated to instructing patients and caregivers on how to accurately complete the on–off diary. The six thematic meetings (Figure 2) were organized in groups of no more than 20 participants (10 patients and 10 caregivers) to allow everyone to actively participate in the lesson and to allow adequate participation in the practical session. Each meeting had a key topic and was divided in a teaching session of 60 min and in a practical session of 60 min with individual training of both, subject and caregiver. To verify comprehension and to ensure adherence, each weekly thematic session was followed by dedicated daily homework (10 min/day for 6 days for patient and caregiver to review accurately the information reported in the filled questionnaires during the day as indicated in practical training of each topic); the homework was collected and reviewed at the following meeting and further clarifications provided if needed.

Every lesson was held by a movement disorders specialist with particular expertise in each field of discussion and the content of each lesson (slides, flyers, questionnaires) was adapted to fit the audience. Neurologists were supported by nurses, physiotherapists and nutritionists when required by the program.

During the meetings, the following topics were discussed:

(1) Motor complications:

Lectures: recognition of the ON–OFF phases; recognition of the difference between dyskinesias, dystonia, akinesia, complicated phases of the disease, recognition of freezing.

Practical training: how to complete an ON–OFF chart and a wearing off questionnaire.

(2) Gastrointestinal disorders:

Lectures: recognition of dysphagia and related feeding strategies; constipation; dietary problems and loss of weight.

Practical training: Motor fluctuations and food; personalized recipes and menu ideas; safety in the kitchen: how to cook safely; training on evacuation diary

(3) Neuropsychological disorders:

Lectures: Insomnia and sleepiness, mood and anxiety, psychosis, behavioral problems.

Practical training: recognition of symptoms and strategies to manage insomnia, daytime sleepiness, depression/anxiety, behavioral disturbances

(4) Autonomic dysfunction:

Lectures: Sweating, hypotension, sexual problems, urinary problems.

Practical training: recognition of non-motor symptoms

(5) Management of drug therapy:

Lectures: medication adherence, side effects, identify a relation between time of treatment intake and motor complications.

Practical training: proper management of drug therapy and side effects, encourage regular medicine intake, medication adherence and motor control

(6) Management rehabilitation therapy:

Lectures: recognition of problems correlated to freezing, postural instability and falls.

Practical training: strategies to manage freezing, postural instability and falls. Training for improving autonomy in activities of daily living; examples of cognitive and instrumental strategies aiming at maintaining the highest level of autonomy.

Pharmacological and non-pharmacological treatment were kept stable throughout the length of the trial (24 weeks) for all subjects involved. Participants requiring any change in medications’ schedule or reporting modifications in their rehabilitation program were early discontinued by the study and evaluated for their final assessments.

### 2.6. Primary outcome

Baseline and post-interventional assessments were performed by movement disorders specialists and neuropsychologists who were not involved in the education program and who were not aware of patients group allocation. The primary outcome was change from baseline to end of School (EoS-6 weeks, T1), 12-weeks FU1 (T2) and 24-weeks FU2 (T3) in the mean daily OFF hours evaluated with the Hauser diary (19). The choice of this primary endpoint was made because we wanted to evaluate the education program as a treatment, therefore choosing the same outcomes that we would use in pharmaceutical clinical trials.

### 2.7. Secondary outcomes

The secondary outcomes were change from baseline to EoS, T2 and T3 in the MDS-UPDRS (20), Hoehn and Yahr (21), MMAS-8 (Morisky Medical Adherence scale-8 items) (22), Beck Depression Inventory (BDI) (23), Montreal Cognitive Assessment (MoCA) (24), Mini Mental State Examination (MMSE) (25), PDQ39 (26), EQ5D (27) (including EQVAS), Caregiver burden inventory (CBI) (28), Instrumental activities of daily living (IADL) (29) and Activities of daily living scale (ADL) (30). The frequency of falls was also investigated as a corollary to the MDS-UPDRS, whenever abnormal scores were reported by patients at the item 2.13 (freezing) and 3.12 (Postural Stability), recording the number of falls 6-months before the baseline (Falls-6 m) reported by patient and during the outpatient visit at T2 (24-weeks).

### 2.8. Statistical analysis

Statistical analyses were conducted with SPSS version 26.0. *t*-Tests and Chi-square tests were performed to compare demographic characteristics and baseline scores of the intervention and the control group; between-group comparisons were performed with one-way ANOVA; a General Linear Model for repeated measure (2-way ANOVA) was used to assess between group comparison on the dependent variables (OFF Time, UPDRS I); within group comparisons were analyzed using paired Student *t*-test. The significance level for all analyses was set to *p* < 0.05. Due to the exploratory nature of the study, no correction was applied for multiple comparisons; *p*-value < 0.05 was considered a statistically significant result. Pearson’s correlation coefficient was used to investigate the linear relationship between OFF time reduction and improvement in functional independence parameters as well as between UPDRS I and QoL assessments. Data were analyzed only if patients and caregiver participated at every meeting. A sensitivity analysis was performed to evaluate the impact of all excluded subjects (screening failures/lost to follow up) on expected outcomes, in the worst-case scenario that all of them did not report any improvement (Δ = 0). The analysis confirmed the significance in favor of the treated group for each outcome, even assuming that the 20 subjects excluded did not report any benefit.

## 3. Results

### 3.1. Study population

A total of 120 patients were screened; 6 patients could not participate (caregiver not available to attend the entire program); 114 were eligible to participate and were randomly assigned to the intervention or control group. In total, 100 patients completed the study, including 52 patients in intervention group and 48 in the control one. Fourteen subjects were lost at the End of School (T1) after randomization, 5 in the intervention and 9 in the control group as reported in the CONSORT flow diagram (Figure 1). In detail, three patients in the intervention group were discontinued, since they did not take part to every meeting, and 2 patients change their pharmacological therapies, therefore were excluded from statistical analysis. Among 9 patients in the control group, 2 were hospitalized (femur fracture and cholecystitis), 4 changed the pharmacological therapy and 3 left the study for caregiver change. At T2 and T3 a total of 5 patients in the intervention group (respectively 3 patients at T2 and 2 patients at T3) and 5 patients in control group (respectively 2 patients at T2 and 3 patients at T3) were withdrawn from the study due to change in their pharmacological therapy (Figure 1).

### 3.2. Baseline characteristics

Patients baseline characteristics and baseline scores for primary and secondary outcomes at baseline were comparable between the groups (Table 1). In particular, among PD patients, no significant difference was observed between the two groups in terms of age, gender, education, disease duration (DD), H&Y, MMSE, number of falls evaluated 6-months before the baseline (FALLS-6 m) and LEDD scores. The same conclusions can be made for patients’ caregivers (Table 1).

**Table 1:** Demographic and baseline characteristics of subjects in the intervention and in the control group.

Patients				Caregivers			
	Cases (*n* = 52)	Control (*n* = 48)	*p*-value		Cases (*n* = 52)	Control (*n* = 48)	*p*-value
Gender				Gender			
Men	32 (61.5%)	24 (50.0%)	0.314	Men	15 (28.8%)	13 (27.1%)	
Women	20 (38.5%)	24 (50.0%)		Women	37 (71.2%)	35 (72.9%)	1.000
Age (years)	68.94 ± 7.0	68.75 ± 8.3	0.900	Age (years)	63.02 ± 11.4	60.88 ± 13.6	0.395
Education				Education			
Low	15 (28.8%)	22 (45.8%)	0.105	Low	20 (38.4%)	13 (27.1%)	0.482
Middle	20 (38.5%)	18 (37.5%)		Middle	21 (40.4%)	23 (47.9%)	
High	17 (32.7%)	8 (16.7%)		High	11 (21.2%)	12 (25.0%)	
DD (years)	11.04 ± 5.07	11.75 ± 4.71	0.470				
H&Y	2.90 ± 0.47	2.88 ± 0.39	0.831				
MMSE	27.08 ± 1.89	27.33 ± 1.78	0.487				
FALLS-6 m	9.25 ± 3.72	9.63 ± 3.22	0.592				
LEDD (mg)	1087.3 ± 212.2	1089.4 ± 229.4	0.964				

One-way ANOVA, *p*-value < 0.05 for Education: low: ≤ 8 years, middle: 9–13 years, high: > 14 years, DD: disease duration; H&Y: Hoehn and Yahr; MMSE: mini-mental state examination; FALLS-6 m: number of falls evaluated 6 months before the baseline; LEDD: Levodopa equivalent daily dose.

### 3.3. Efficacy outcomes

At the end of School, patients in the intervention group showed a significant reduction in daily OFF hours (from 3.32 ± 1.50 to 2.24 ± 1.39) compared to control patients, who reported instead an increase in daily OFF hours (from 3.12 ± 1.38 to 3.21 ± 1.44), *p* = 0.0009, as indicated in Table 2. In the treatment group, the mean change in the primary endpoint (hours daily OFF time) from baseline to EoS was highly significant [−1.07 (SD 0.78) vs. + 0.09 (SD 0.35), *p* < 0.0001] (Table 3). Moreover, the percentage of subjects that reported this improvement was much higher than expected and extremely significant [82.7% vs. 8.3%, *χ*^2^ = (1, *N* = 100) = 55.404, *p* < 0.0001]. In the intervention group, the MDS-UPDRS part I, which analyses the non-motor aspects of experiences of daily living, improved significantly (*p* = 0.005) as well as the measures of functional independence (IADL and ADL: *p* = 0.005 and *p* = 0.024 respectively) and the MMAS-8 (8.69 ± 1.43 vs. 7.46 ± 1.53 at EoS, *p* < 0.0001) with respect control group.

**Table 2:** Baseline and end of school (EoS) outcome measures for all participants and comparison between groups.

Outcomes		Baseline			EoS-T1			Cases (*n* = 52)	Control (*n* = 48)	*p*-value	Cases (*n* = 52)	Control (*n* = 48)	*p*-value
Motor	OFF time	3.32 ± 1.50	3.12 ± 1.38	0.484	2.24 ± 1.39	3.21 ± 1.44	0.0009
	MDS-UPDRS I	14.85 ± 4.63	14.48 ± 4.87	0.700	12.42 ± 4.01	15.00 ± 4.86	0.005
	MDS-UPDRS II	15.71 ± 9.06	14.48 ± 5.82	0.425	14.37 ± 6.81	15.31 ± 6.34	0.475
	MDS-UPDRS III	27.21 ± 10.19	23.85 ± 7.09	0.061	26.94 ± 9.82	24.13 ± 7.66	0.115
	MDS–UPDRS IV	6.69 ± 2.44	5.85 ± 2.02	0.065	5.87 ± 2.22	5.90 ± 2.14	0.945
Cognitive/psychiatric	MoCA	25.02 ± 3.97	25.58 ± 2.96	0.426	25.60 ± 3.63	25.38 ± 3.01	0.742
	BDI	13.38 ± 4.77	11.96 ± 6.24	0.200	12.29 ± 4.67	12.25 ± 5.93	0.971
Functional independence	IADL	3.96 ± 1.38	4.23 ± 1.07	0.286	4.81 ± 1.28	4.13 ± 1.06	0.005
	ADL	3.15 ± 1.26	3.63 ± 1.35	0.074	4.00 ± 1.42	3.38 ± 1.28	0.024
QoL	EQVAS	52.79 ± 15.54	57.50 ± 12.59	0.101	60.58 ± 14.33	55.10 ± 13.23	0.051
	EQ5D	8.69 ± 1.90	8.27 ± 1.99	0.282	7.92 ± 1.96	8.19 ± 2.13	0.519
	PDQ 39	59.81 ± 25.28	56.58 ± 22.84	0.506	53.02 ± 21.23	57.58 ± 23.44	0.309
Treatment adherence	MMAS-8	6.83 ± 1.38	7.31 ± 1.53	0.099	8.69 ± 1.43	7.46 ± 1.53	<0.0001
Caregiver burden	CBI	22.81 ± 15.56	24.17 ± 16.44	0.672	19.15 ± 13.53	24.83 ± 16.47	0.062

One-way ANOVA, *p*-value < 0.05.

**Table 3:** Baseline and EoS outcome measures for all participants and comparison within groups.

Outcomes		Cases (*n* = 52)	*p*-value	Control (*n* = 48)	*p*-value
Motor	*Primary*				
	Off time (h)	−1.07 (±0.78)	<0.0001	0.09 (±0.35)	0.071
	*Secondary*				
	HY	−0.009 (±0.07)	0.322	0.05 (±0.18)	0.058
	UPDRS I	−2.42 (±2.35)	<0.0001	0.52 (±1.43)	0.015
	UPDRS II	−1.35 (±5.55)	0.087	0.83 (±2.25)	0.014
	UPDRS III	−0.27 (±1.07)	0.075	0.27 (±1.48)	0.212
	UPDRS IV	−0.83 (±1.18)	<0.0001	0.04 (±0.54)	0.598
Cognitive/Psychiatric	MoCA	0.58 (±0.89)	<0.0001	−0.21 (±0.74)	0.058
	BDI II	−1.10 (±2.60)	0.004	0.29 (±1.30)	0.128
Functional independence	IADL	0.85 (±0.77)	<0.0001	−0.10 (±0.55)	0.200
	ADL	0.85 (±0.70)	<0.0001	−0.25 (±0.56)	0.004
QoL	PDQ 39	−6.79 (±10.58)	<0.0001	1.00 (±6.77)	0.312
	EQ5D	−0.77 (±1.08)	<0.0001	−0.08 (±0.71)	0.420
	EQVAS	7.79 (±9.04)	<0.0001	−2.39 (±9.22)	0.078
Treatment adherence	MMAS-8	1.86 (±1.12)	<0.0001	0.15 (±0.85)	0.241
Caregiver burden	CBI	−3.65 (±4.28)	<0.0001	0.67 (±2.24)	0.045

Paired Student *t*-test, *p*-value < 0.05.

When comparing the changes between baseline and EoS values within groups, a significant improvement was also noted in non-motor symptoms and motor complications (MSD-UPDRS I and IV), in cognitive and mood domains, QoL and in parameter of functional independence (Table 3).

In the control group a statistically significant deterioration was noted for MDS-UPDRS part I and II concerning both non-motor and motor experiences of daily living and for one of the functional independence parameters [ADL: − 0.25 (± 0.56) p 0.004].

The sensitivity analysis performed did not affect the results, confirming the significance obtained in each outcome.

Of the 100 caregivers analyzed 28 were men and 72 women, with no significant difference in gender representation in the two groups (Table 1). As expected, there was a prevalence of female caregivers in both groups, but no relationship was found between the number of women and the CBI outcome in the intervention group (*F* = 3.015, *p* = 0.089). In the intervention group caregiver burden diminished considerably [−3.65 (± 4.28), *p* < 0.0001] while the control group showed a significant worsening [0.67 (± 2.24), *p* = 0.045] (Table 2).

**Figure 3:**
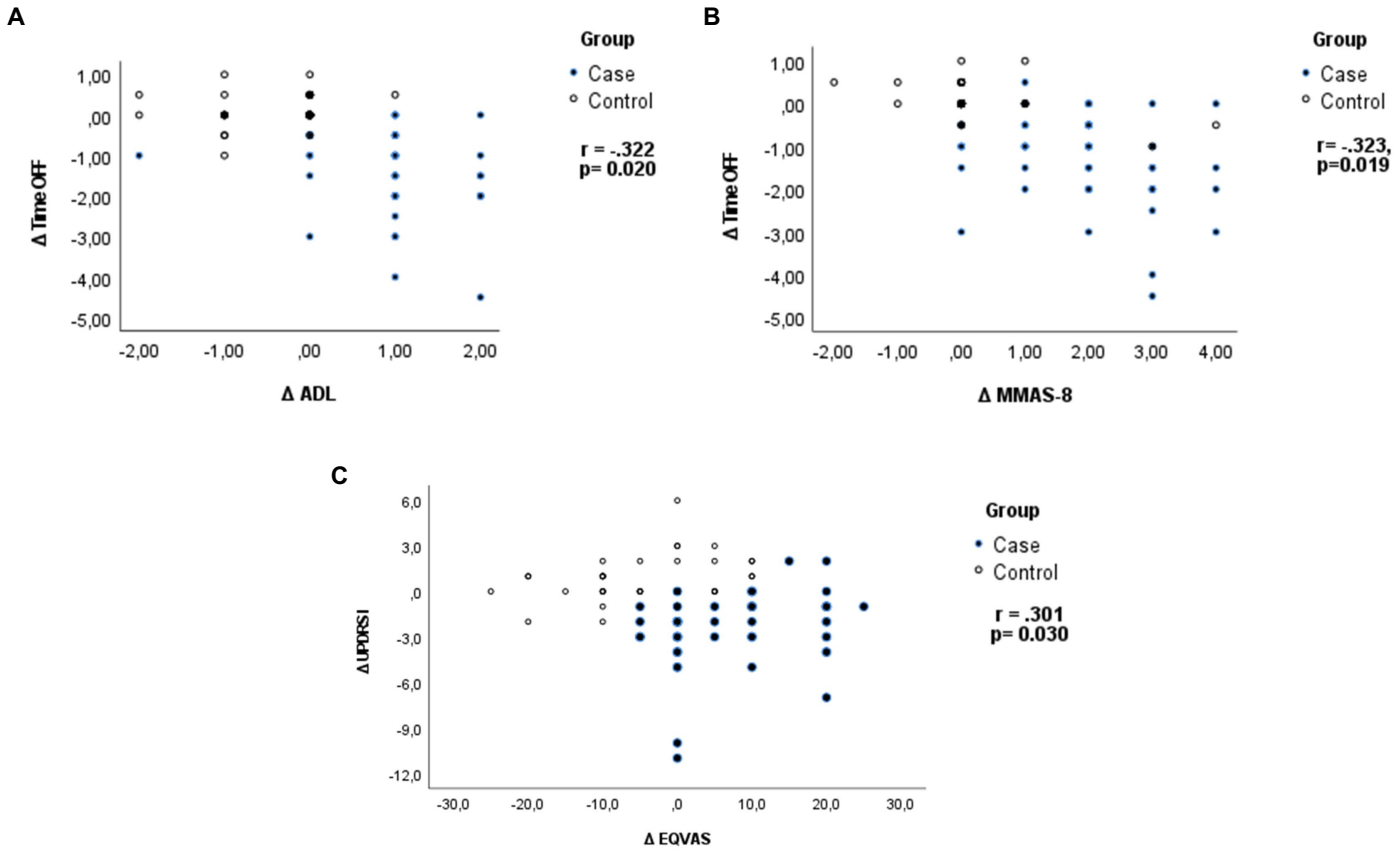
Pearson’s correlation coefficient: **(A)** Analysis of relation between OFF time reduction and the improvement in functional independence (ADL) and **(B)** medications adherence (MMAS-8), and **(C)** between the improvement in UPDRS I and Δ EQVAS.

A significant correlation was found between OFF time reduction and the improvement at the ADL (*r* = −0.322, *p* = 0.020) and MMAS-8 (*r* = −0.323, *p* = 0.019) and between the improvement of the UPDRS I and the Δ EQVAS (*r* = 0.301, *p* = 0.030, respectively; Figure 3). The level of education was found not to affect the response of the patients treated compared to that of the controls (*χ*^2^ = 4,517; dof = 2 *p* = 0.105). Moreover, a one-way *post hoc* with Bonferroni was performed to investigate the relation between education level and outcomes: the only found was ΔUPDRS III in the control group, which showed a significant difference between the middle and the high category: 1.58, *p* = 0.034.

At the 12-(T2) and 24-weeks (T3) follow-up, despite a slight decrease compared to 6-weeks EoS, the interventional group maintained the improvement in daily OFF time with respect to the control group (2.40 ± 1.40 vs. 3.11 ± 1.40, *p* = 0.015 and 2.50 ± 1.42 vs. 3.14 ± 1.46, *p* = 0.038, respectively, at 12- and 24-weeks FU). Furthermore, the difference in daily OFF time and the therapy adherence remained statistically significant between groups at 12- and 24-weeks follow-up (Table 4). Interestingly, a significant decrease in the fall rate was observed between the groups (8.13 ± 3.33 vs. 9.98 ± 3.60, *p* = 0.013) and within groups at 24-weeks follow-up compared to fall rate in the previous 6-months the baseline (Tables 4, 5).

**Table 4:** Twelve-weeks (T2) and 24-weeks (T3) follow-up outcome measures for all participants and comparison between groups.

Outcomes	Follow up 12-weeks (T2)			Follow up 24-weeks (T3)			Cases (*n* = 49)	Control (*n* = 46)	*p*-value	Cases (*n* = 47)	Control (*n* = 43)	*p*-value
*Primary*						
OFF time (h)	2.40 ± 1.40	3.11 ± 1.40	0.015	2.50 ± 1.42	3.14 ± 1.46	0.038
						
*Secondary*						
UPDRS III	26.86 ± 9.89	24.46 ± 7.32	0.184	26.64 ± 9.54	24.37 ± 7.40	0.214
MOCA	25.84 ± 4.67	25.54 ± 2.86	0.715	25.81 ± 4.75	25.58 ± 2.94	0.788
H&Y	2.91 ± 0.48	2.90 ± 0.39	0.852	2.90 ± 0.45	2.91 ± 0.38	0.976
MMSA-8	8.71 ± 1.41	7.48 ± 1.55	<0.0001	8.70 ± 1.46	7.47 ± 1.56	0.0002
FALLS-6 m	-	-	-	8.13 ± 3.33	9.98 ± 3.60	0.013
CBI	19.65 ± 13.97	25.15 ± 16.93	0.087	19.64 ± 14.18	24.40 ± 15.63	0.134

One-way ANOVA, *p*-value < 0.05.

**Table 5:** Six-weeks (T1), 12-weeks (T2) and 24-weeks (T3) follow-up outcome measures for all participants and comparison with baseline (T0) within the intervention group.

	Cases EoS 6-weeks (T1) (*n* = 52)	*p*-value	Cases T2 (FU-12 weeks) (*n* = 49)	*p*-value	Cases T3 (FU-24 weeks) (*n* = 47)	*p*-value
*Primary outcome*
Off time (h)	−1.07 (±0.78)	<0.0001	−0.90 (±0.65)	<0.0001	−0.76 (±0.59)	<0.0001
*Secondary outcomes*
HY	−0.009 (±0.07)	0.032	−0.010 (±0.07)	0.322	−0.032 (±0.12)	0.083
MoCA	0.58 (±0.89)	<0.0001	0.71 (±2.96)	0.089	0.79(±3.08)	0.071
UPDRS III	−0.27 (±1.07)	0.075	−0.43 (±1.00)	0.0043	−0.13(±1.06)	0.411
MMSA-8	1.85 (±1.09)	<0.0001	1. 90(±1.07)	<0.0001	1.85 (±1.04)	<0.0001
FALLS 6 m	–	–	–	–	−0.89 (±1.18)	<0.0001
CBI	−3.65 (±4.28)	<0.0001	−3.14 (±4.16)	<0.0001	−2.62 (±3.64)	<0.0001

Paired Student *t*-test, *p*-value < 0.05.

Moreover, the comparison between baseline and T2 and T3 follow-u*p* values within groups revealed that the intervention group maintained the significant improvement in most secondary outcomes analyzed (Table 5). Alongside, in the control group a statistically significant deterioration in stage disease (H&Y at 24-weeks FU), in motor symptoms (MDS-UPDRS part III at 12- and 24-weeks FU) and fall rate have to be underlined (Table 6). Concerning the caregiver burden, CBI data at 12- and 24-weeks follow-up remark how the education therapy may positively affect the caregiver daily life (Tables 5, 6).

**Table 6:** Six-weeks (T1), 12-weeks (T2) and 24-weeks (T3) follow-up outcome measures for all participants and comparison with baseline (T0) within the control group.

	Control EoS 6-weeks (T1) (*n* = 52)	*p*-value	Control T2 (FU-12 weeks) (*n* = 49)	*p*-value	Control T3 (FU-24 weeks) (*n* = 47)	*p*-value
*Primary outcome*
Off time (h)	0.09 (±0.35)	0.071	−0.01 (±0.07)	0.322	0.10 (±0.37)	0.071
*Secondary outcomes*
HY	0.05 (±01.19)	0.058	0.01 (±0.07)	0.322	0.07 (±0.21)	0.032
MoCA	−0.21 (±0.74)	0.058	−0.15 (±0.52)	0.051	−0.12 (±0.39)	0.058
UPDRS III	0.27 (±1.48)	0.212	0.91 (±1.46)	0.0001	1.23 (±1.59)	<0.0001
MMSA-8	0.15 (±0.89)	0.241	0.13 (±0.88)	0.323	0.12 (±0.82)	0.360
FALLS 6 m	–	–	–	–	0.63 (±0.79)	<0.0001
CBI	0.67 (±2.24)	0.045	1.15 (±2.16)	0.006	1.56 (±2.32)	<0.0001

Paired Student *t*-test, *p*-value < 0.05.

**Figure 4:**
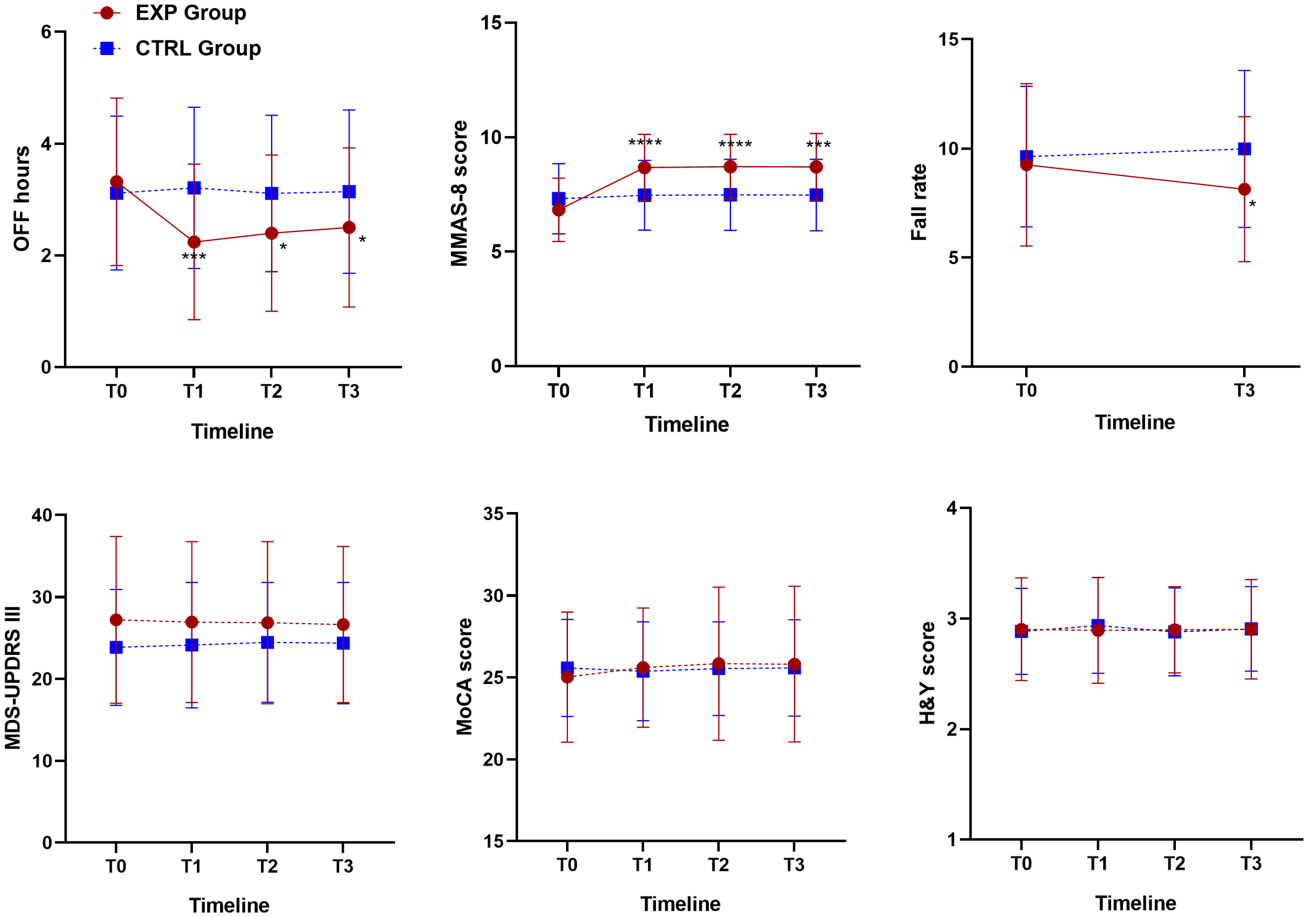
Motor and non-motor outcome measures of education therapy: OFF hours, MMAS-8, UPDRS III, MoCA and H&Y at baseline (T0), 6-weeks (T1, EoS), 12-weeks (T2) and 24-weeks (T3) follow-up for all participants and comparison between intervention and control groups. One-way ANOVA, *p*-value <0.05. For fall rate, outcome measures of education therapy referred to 6-months before the baseline assessment (T0, Falls-6 m) and 24-weeks FU (T3). One-way ANOVA, *p*-value < 0.05. According to GraphPad Prism 7 software, ^*^ *p*-value from 0.01 to 0.05 (Significant), ^***^ *p*-value from 0.0001 to 0.001 Extremely significant, ^****^ *p*-value <0.0001 extremely significant.

Motor and non-motor outcome measures of education therapy were plotted in Figure 4, in which the mean and SD of OFF hours, MMAS-8, UPDRS III, MoCA and H&Y at baseline (T0), 6-weeks (T1, EoS), 12-weeks (T2) and 24-weeks (T3) follow-up, for all participants were reported and compared between intervention and control groups.

### 3.4. Discussion

The need for education programs has been recently reinforced by a survey among movement disorders specialists and general neurologists across the United States (31). The study evidenced a gap between doctors and patients’ notion of motor and non-motor symptoms, treatment complication and side effects which made periodic visits sometimes ineffective. The neurologists interviewed highlighted the need for “education materials, techniques and patients’ self-management tools” to facilitate clinical communication as well as the development of “strategies for eliciting non-motor symptoms, motor complications and contextualizing symptoms,” reaffirming that the best route to optimize clinical encounter and increase compliance is education.

In this regard, the results of our study show the value of education therapy in supporting and integrating medical treatment and physiotherapy in PD management. The innovative contribution of the study resides in its demonstration of the efficacy of this education program on non-motor experiences of daily living, as highlighted by the improvement in MDS-UPDRS I at 6-weeks End of School. Most crucially, in the intervention group, 82.69% of patients showed a reduction in the time spent in OFF with an average of −1.07 (±0.78) OFF hours per day, while the control group showed a significant worsening trend. It must be noted that the reduction in daily OFF time is the most used clinical endpoint in clinical trials for Parkinson’s disease with motor fluctuations. All successful PD medications have demonstrated a treatment effect of more than 1 h reduction of daily OFF time (32). The rate of improvement observed in this study is similar of what obtained with the majority of levodopa adjunct treatments, demonstrating the important contribution of education therapy in reducing patient’s disability. Among previous studies analyzing the efficacy of education programs, the reduction in daily off time was investigated exclusively by Marumoto et al. (33), who reported a reduction in the average OFF time per day of 0.26 h. It should be considered though that this study evaluated a combined protocol of rehabilitation and education therapy, comparing it with the rehabilitation treatment only, and that the mean OFF time of the two groups at baseline was considerably lower (0.58 h). The improvement in motor fluctuations evidenced in this study is likely related to the improved drug compliance reached through the education program. Indeed, a significant improvement in therapeutic adherence assessed with the MMAS-8 scale was found [1.86 (± 1.12), *p* < 0.0001] which positively correlated with the reduction in daily OFF time. It can be assumed that a greater understanding of the mechanisms of action of antiparkinsonian drugs, and especially levodopa, had a crucial role in determining the outcome, as highlighted from the disappearance of the post-prandial OFF reported by most patients after the second meeting, in which the interference between meals and levodopa was explained. Another important goal reached with this education program was the amelioration of subjects’ compliance. Patients with chronic conditions and complex drug regimens are known to be at high risk of poor compliance (34). PD education programs may overcome this issue increasing awareness of the pathology and its treatments, allowing patients to regain control over the disease, feeling that they no longer adhere to other people’s decisions or imposed therapies, but that they have acquired an active role in the management of their condition.

The improvement of MDS-UPDRS I and II has already been reported following PD education programs (16). This result is particularly important since it shows a significant improvement in non-motor symptoms which still represent a therapeutic challenge for their high prevalence and their negative impact on disease and QoL (35). According to a recent survey, 48% of PD patients and 58% of caregivers reported a greater impact on QoL of non-motor symptoms compared to motor symptoms. Furthermore, all participants affirmed the importance of receiving more information about these symptoms and their management (36), knowledge that can be easily reached with patients/caregiver education.

Among secondary outcomes, a statistically significant improvement was observed in all QoL scales and the BDI-II between baseline and EoS in the intervention group. The improvement in the quality-of-life scales has been already reported in previous studies on education programs (14, 16) while no study had shown improvements in depressive symptoms. It must be taken into account though that different rating scales were used in the various studies and that most of them were judged too short to have a major impact on mood (16) even if a general impression of improvement was reported by the majority of participants (14–17).

Finally, in the intervention group, the caregiver burden, evaluated through the CBI, resulted in a statistically significant improvement, which positively correlated with the reduction in the amount of daily OFF time and the improvement in patient’s functional independence (ADL). The results obtained are in line with what already reported in literature (15) but this is the first study that linked the reduction in caregiver burden directly to the improvement of motor condition and not solely to the management of psycho-social issues.

The improvement in treatment adherence was retained up to 24 weeks after the end of the education program, with a positive effect on both the total daily OFF time and the caregiver burden. The analysis performed at T2 and T3 confirmed the longstanding beneficial effect of the education therapy on the primary outcome (daily OFF time), and on some of the secondary outcomes (MDS-UPDRS III and fall rate).

It must be taken into account that the participation in the program may have triggered a feeling of being special and uniquely monitored and be associated with a possible placebo effect. Also, the study did not include a “sham” intervention for the control group, with equal number of appointments but without the education component, which can be considered the main limitation of this study. It must be acknowledged though that the primary outcome chosen for the study, i.e., change in the mean daily OFF hours, is less susceptible to a placebo effect than QoL or ADL measures and that the improvement observed at the End of School was maintained up to 6 months after the end of the intervention, limiting the possibility that a placebo effect may have played a role in such change.

A cost analysis of the education program was not in the scope of this study and therefore a formal cost-effectiveness analysis was not performed. Since the Italian National Health System (NHS) does not yet contemplate an education program for Parkinson’s disease, it is plausible to consider the education therapy for diabetes, currently available, as model for a cost estimation. Assuming the same fees would have been applied, the cost for an education therapy for Parkinson’s disease may be absolutely affordable for both, the NHS and patients.

### 3.5. Conclusion

The results obtained in the present study demonstrated that education programs may translate in a notable improvement in motor fluctuations and non-motor symptoms in advanced patients with PD. The important enhancement in functional independence confirms the effectiveness of this type of programs in ameliorating patients’ quality of life. Education programs increase patient knowledge and awareness, ameliorate confidence in the decision-making process and patient-doctor interaction which translates into a positive attitude and greater compliance with treatment.

## Data availability statement

The raw data supporting the conclusions of this article will be made available by the authors, without undue reservation.

## Ethics statement

The studies involving human participants were reviewed and approved by San Raffaele Ethic Committee and ASL Rome II Ethic Committee. The patients/participants provided their written informed consent to participate in this study.

## Author contributions

MT, RR, LI, MLDV, MC, VS, and SP contributed to the data collection and analysis. MT, LV, FV, MFDP, and FS contributed in the supervision of the research study. MFDP, MT, RR, LI, MLDV, MC, LV, FV, VS, SP, and FS contributed to the design of the study, to draft and review the manuscript. All authors have read and approved the final manuscript.

## Funding

This study was partially funded by the Socialmente 2 Call, promoted by the Service Centre for Volunteering of the Lazio Region (CESV), by the Italian Ministry of Health (Ricerca Corrente RC-2022) to MC, LV, SP, FS IRCCS San Raffaele and by San Raffaele Cassino to RR and MFDP. This education project was inspired by the Parkinson Ciociaria Association and co-funded by third sector and local government partners and was conducted with the partnership of San Raffaele Cassino and IRCCS San Raffaele Roma and San Giovanni Battista Hospital, Rome (Italy).

## Conflict of interest

The authors declare that the research was conducted in the absence of any commercial or financial relationships that could be construed as a potential conflict of interest.

## Publisher’s note

All claims expressed in this article are solely those of the authors and do not necessarily represent those of their affiliated organizations, or those of the publisher, the editors and the reviewers. Any product that may be evaluated in this article, or claim that may be made by its manufacturer, is not guaranteed or endorsed by the publisher.

## Footnotes

^1^ https://clinicaltrials.gov/ct2/show/NCT04378127
